# Pretreatment glucose status determines HCC development in HCV patients with mild liver disease after curative antiviral therapy

**DOI:** 10.1097/MD.0000000000004157

**Published:** 2016-07-08

**Authors:** Chung-Feng Huang, Ming-Lun Yeh, Cing-Yi Huang, Pei-Chien Tsai, Yu-Min Ko, Kuan-Yu Chen, Zu-Yau Lin, Shinn-Cherng Chen, Chia-Yen Dai, Wan-Long Chuang, Jee-Fu Huang, Ming-Lung Yu

**Affiliations:** a Hepatobiliary Division, Department of Internal Medicine, Kaohsiung Medical University Hospital, Kaohsiung Medical University, Kaohsiung, Taiwan; b Department of Occupational Medicine, Kaohsiung Municipal Ta-Tung Hospital, Kaohsiung Medical University, Kaohsiung, Taiwan; c Faculty of Internal Medicine, School of Medicine, College of Medicine, Kaohsiung Medical University, Kaohsiung, Taiwan; d Health Management Center, Kaohsiung Medical University Hospital, Kaohsiung Medical University, Kaohsiung, Taiwan; e Center for Infectious Disease and Cancer Research, Kaohsiung Medical University, Kaohsiung, Taiwan; f Institute of Biomedical Sciences, National Sun Yat-Sen University.

**Keywords:** DM, HCC, HCV, OGTT, Pre-DM, SVR, treatment

## Abstract

Although diabetes mellitus (DM) is known to increase the risk of hepatitis C virus (HCV)-related hepatocellular carcinoma (HCC), the impact of dynamic glucose status on HCC occurrence in chronic hepatitis C (CHC) patients receiving antiviral therapy is unclear. In total, 1112 biopsy-proven patients treated with peginterferon/ribavirin were enrolled in this study. Both pretreatment and post-treatment glucose status, including 75 g oral glucose tolerance test (OGTT), were measured to evaluate the association between glucose status and the development of HCC. Of the 1112 patients evaluated, 93 (8.4%) developed HCC >5183.8 person-years of follow-up (annual incidence rate: 1.79%). DM only influenced the risk of developing CC in patients with mild liver disease (F0-2) and a sustained virological response (SVR) but not in other patient subpopulations. Cox-regression analysis demonstrated that the strongest factor associated with HCC in patients with mild liver disease and SVR was the presence of DM (hazard ratio [HR]/95 % confidence intervals [CI]: 3.79/1.420–10.136, *P* = 0.008), followed by age (HR/CI: 1.06/1.001–1.117, *P* = 0.046) and platelet count (HR/CI: 0.989/0.979–1.000, *P* = 0.05). The percentages of SVR patients with F0-2 with normoglycemia, pre-DM, sub-DM (pre-sDM), and DM before treatment were 45.3% (n = 267), 29.9% (n = 176), 15.6% (n = 92), and 9.2% (n = 54), respectively. The percentages of HCC in patients with normoglycemia, pre-sDM, and DM were 1.1%, 3.7%, and 11.1%, respectively (trend *P* < 0.001). Sixteen of the 19 (84.2 %) HCC patients possessed glucose abnormality (including 6 patients with DM and 10 patients with pre-sDM) before antiviral therapy. Compared to patients with normoglycemia, the incidence of HCC increased gradually from pre-sDM (HR: 3.6, *P* = 0.05) to DM (HR: 11.6, *P* = 0.001) (adjusted trend *P* = 0.004). We concluded that DM is a critical determinant for the development of HCC in SVR patients with mild liver disease. Pre-sDM status carried an additional risk for HCC, and these patients should also be carefully monitored for HCC after viral eradication.

## Abstract 2

Supplemental Digital Content is available in the text

## 1 Introduction

Several genetic and environmental factors as well as occupational exposure to carcinogenic toxic substances may lead to hepatocellular carcinoma (HCC).^[1]^ Hepatitis C virus (HCV) infects ∼180 million people, and it is one of the leading causes of HCC worldwide. The seroprevalence of HCV infection has been increasing over the past few decades,^[2,3]^ and as a consequence, the burden of HCC-related to HCV has been growing more rapidly than other etiologies of liver disease.^[4,5]^

There is a strongly mutual linkage between HCV and diabetes mellitus (DM), and DM is an important feature of the extrahepatic manifestationsof HCV infection.^[6]^ The presence of DM determines the disease activity, disease course, and clinical outcomes of HCV.^[7]^ Although the association of DM with HCC is controversial, particularly in hepatitis B virus (HBV) and HCV endemic areas,^[8,9]^ recent meta-analyses has clearly demonstrated that CHC patients with DM carry a higher risk of developing HCC than those without DM.^[10]^ There is also emerging evidence that certain antidiabetic drugs may modify the risk of HCC development.^[11]^ Based on the causal relationship between DM and HCV, it has been suggested that the contributory risk of DM for HCC development is higher in patients with chronic hepatitis C (CHC) than HBV-infected subjects or those without HBV and HCV infections.^[12]^ On the other hand, glucose status is associated with the antiviral treatment outcome of CHC infection. The presence of DM or insulin resistance is regarded as an unfavorable predictor of treatment efficacy in patients receiving interferon-based therapy.^[13]^ Meanwhile, elevations in glucose status may be ameliorated after curative antiviral therapy.^[14,15]^ Although successful HCV eradication with antiviral therapy is known to reduce the risk of HCC in patients with all stages of liver disease,^[16,17]^ the effect of DM in HCC among HCV treatment cohorts is less clear.^[18]^ Importantly, it has been demonstrated that prediabetes increases the risk of cardiovascular disease.^[19]^ The risk of HCC development in CHC patients with different glucose statuses is unknown, and the impact of glucose status amelioration on HCC occurrence after antiviral treatment has never been explored. Therefore, we evaluated the influence of pre- and post-treatment glucose status on HCC development after longitudinal follow-up in a large cohort of CHC patients who had received antiviral therapy.

## 2 Methods

CHC patients receiving antiviral therapy (either peginterferon alfa-2a or peginterfer on alfa-2b plus ribavirin) were consecutively recruited as a prospective follow-up cohort at 1 tertiary hospital and 2 core regional hospitals from 2001 to 2012. Patients were excluded if they were coinfected with HIV or HBV. Patients were also excluded if they abused alcohol (≥20 g daily) or had evidence of HCC before, during, or within 6 months postantiviral therapy.

Patients who did or did not achieve a sustained virological response (SVR), defined as seronegativity for HCV RNA throughout a 24-week post-antiviral treatment follow-up period, were evaluated further for the risk of HCC development. The post-treatment follow-up strategy was based on cirrhotic status and treatment outcome, as previously described.^[16]^ In general, patients were followed up at least every 3 months if they had advanced liver disease or did not achieve SVR and at least every 6 to 12 months if they had mild liver disease and achieved SVR. The diagnosis of HCC was confirmed by histology or on the basis of image and laboratory evidence, as defined by the American Association for the Study of Liver Diseases^[20]^ and Asian Pacific Association for the Study of the Liver^[21]^ guidelines.

Serum HCV RNA was detected using qualitative real-time polymerase chain reaction (PCR) (COBAS AMPLICOR Hepatitis C Virus Test, ver. 2.0; Roche, Branchburg, NJ, detection limit: 50 IU/mL) and quantification branched DNA assay (Versant HCV RNA 3.0, Bayer, Tarrytown, NJ; quantification limit: 615 IU/mL) before 2011. The HCV genotypes were determined using the Okamoto method before 2011.^[22]^ Both HCV RNA and genotype were detected using real-time PCR assay (Real Time HCV; Abbott Molecular, Des Plaines, IL; detection limit: 12 IU/mL) since 2011.^[23]^ Liver disease severity was evaluated by liver biopsy, and histology was graded and staged according to the scoring system described by Scheuer.^[24]^ To prevent the potential pitfall of sampling variability in liver biopsy, we evaluated the association of the risk factors with HCC by stratifying patients according to disease severity: mild (F0-2) or advanced (F3-4).^[7]^ All patients provided written informed consent. The institutional review board at the participating hospital approved the protocols, which conformed to the guidelines of the International Conference on Harmonization for Good Clinical Practice.

### 2.1 Definition of glucose status

DM history and coadministration of oral hypoglycemic agents or insulin were reviewed by the physicians and recorded by trained coordinators in the outpatient department before, during, and after antiviral therapy. Because glucose abnormalities might be underestimated by measuring fasting plasma glucose (FPG) alone, particularly in CHC patients,^[25]^ 75 g oral glucose tolerance tests (OGTT) were performed in patients without DM history before and 6 months after completing treatment, as previously described.^[15,26]^ The judgment of glucose abnormality was based on the definition established by the American Diabetes Association.^[27]^ Briefly, patients were categorized as having known DM if FPG levels were >126 mg/dL or HbA1C was >6.5 %at least twice in the medical record, if there was a previously established diagnosis of DM, or if the patient was currently taking any form of hypoglycaemic drugs or insulin injections. Impaired fasting sugar (IFG) was diagnosed if the FPG was between 100 and 125 mg/dL. Impaired glucose tolerance (IGT) was diagnosed according to a 2-hour plasma glucose concentration of 140 to 199 mg/dL. Patients with IFG, IGT, or HbA1C between 5.7% and 6.4% were defined as cases of prediabetes (pre-DM). For patients without known DM, subclinical DM (Sub-DM) was diagnosed if they met DM criteria with OGT Tresult (2-hplasma glucose concentration of ≥200 mg/dL).

### 2.2 Statistical analyses

Frequency was compared between groups using the χ^2^ test with the Yates correction or Fisher's exact test. Group means (presented as the mean ± standard deviation) were compared using analysis of variance and Student's *t* test or the nonparametric Mann–Whitney test when appropriate. Kaplan–Meier analysis and the log-rank test were performed by comparing the differences of the cumulative incidence of HCC between determinants. The risk factors independently associated with HCC development were evaluated using Cox regression analysis. The statistical analyses were performed using the SPSS 12.0 statistical package (SPSS, Chicago, IL). All statistical analyses were based on 2-sided hypothesis tests with a significance level of *P* < 0.05.

## 3 Results

### 3.1 Patient profile

A total of 1112 patients were enrolled in the current study, with a median follow-up period of 55.9 months (range: 6–142 months). The demographic, clinical, and virological features at base line are shown in Table 1. The mean age of the patients was 52.1 years, and 52.8% of the patients were male. A total of 397 (35.7%) patients had advanced liver fibrosis (F34), and 863 (77.6%) achieved SVR after antiviral therapy.

**Table 1:**
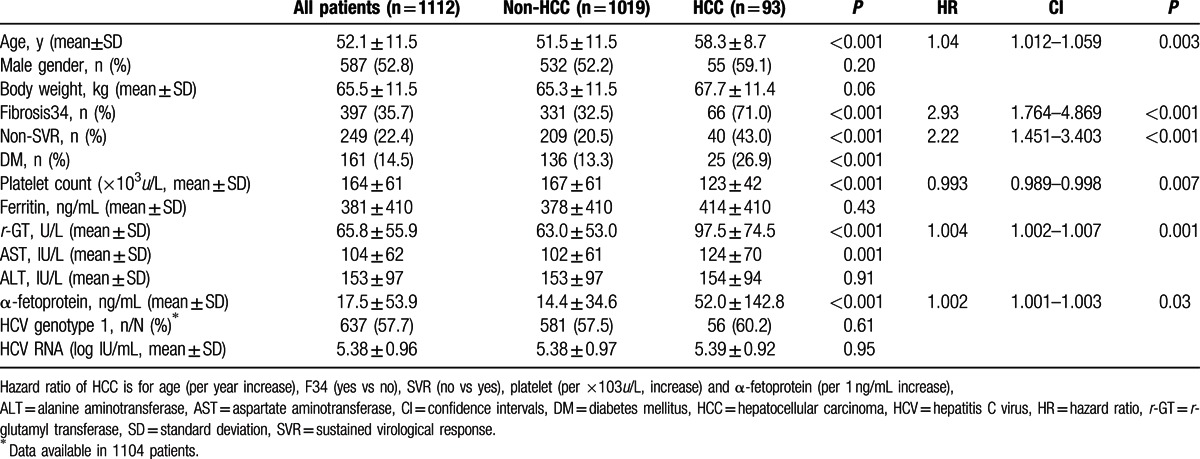
Factors associated with HCC development of the entire cohort.

### 3.2 Risk factors for HCC development

Of the 1112 patients analyzed, 93 (8.4%) developed HCC >5183.8 person-years of follow-up (annual incidence rate: 1.79 %). Patients who developed HCC were older and had a higher incidence of advanced liver fibrosis; a lower SVR rate; lower platelet counts; and higher levels of aspartate aminotransferase (AST), *r-*glutamyl transferase (*r*-GT) andα-fetoprotein (AFP). Cox-regression analysis revealed that the independent factor most strongly associated with HCC in the treatment cohort was advanced liver disease (hazard ratio [HR]/ 95 % confidence intervals [CI]: 3.22/1.959–5.298, *P* < 0.001), followed by non-SVR (HR/CI: 2.23/1.462–3.394, *P* < 0.001), old age (HR/CI: 1.04/1.013–1.061, *P* = 0.002), low platelet count (HR/CI: 0.993/0.989–0.998, *P* = 0.006), and high *r*-GT (HR/CI: 1.004/1.002–1.007, *P* = 0.001) and AFP levels (HR/CI: 1.002/1.001–1.004, *P* = 0.002) (Table 1). DM was not a risk factor for developing HCC after adjusting for other potential confounders.

### 3.3 Role of DM in HCC development in patients with differing liver disease severity and treatment outcome

**Figure 1:**
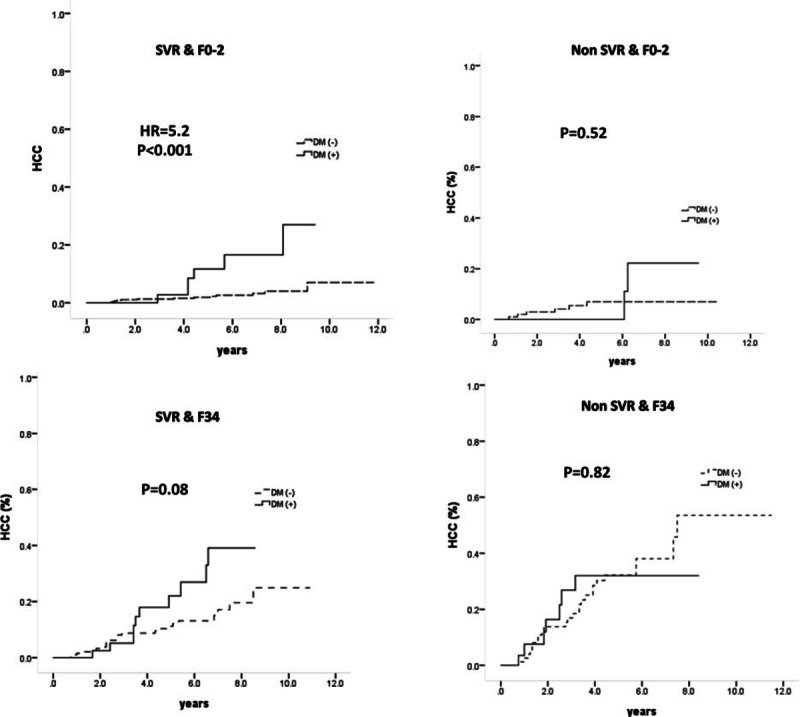
HCC development in patients stratified by liver disease severity and SVR status. HCC = hepatocellular carcinoma, SVR = sustained virological response.

Because advanced liver fibrosis and failure to attain SVR were the major determinants for HCC, we further analyzed the association between DM and HCC by stratifying patients according to these 2 major risk factors. As shown in Fig. 1, DM influenced the occurrence of HCC in patients with mild liver disease (F0-2) and SVR but not the other 3 subpopulations examined. For SVR patients with mild liver disease who had DM, the 1-, 3-, and 5-year cumulative incidence rates of HCC were 0%, 2.8%, and11.7%, respectively, whereas the cumulative incidence rates for patients without DM were 0.2%, 1.3%, and 1.9%, respectively (HR 5.2, 95% CI: 1.97–13.69, *P* < 0.001). Cox regression analysis revealed that the strongest predictive factor for HCC in SVR patients with mild liver disease was the presence of DM (HR/CI: 3.79/1.420–10.136, *P* = 0.008), followed by age (HR/CI: 1.06/1.001–1.117, *P* = 0.046) and platelet count (HR/CI: 0.989/0.979–1.000, *P* = 0.05) (Table 2).

**Table 2:**
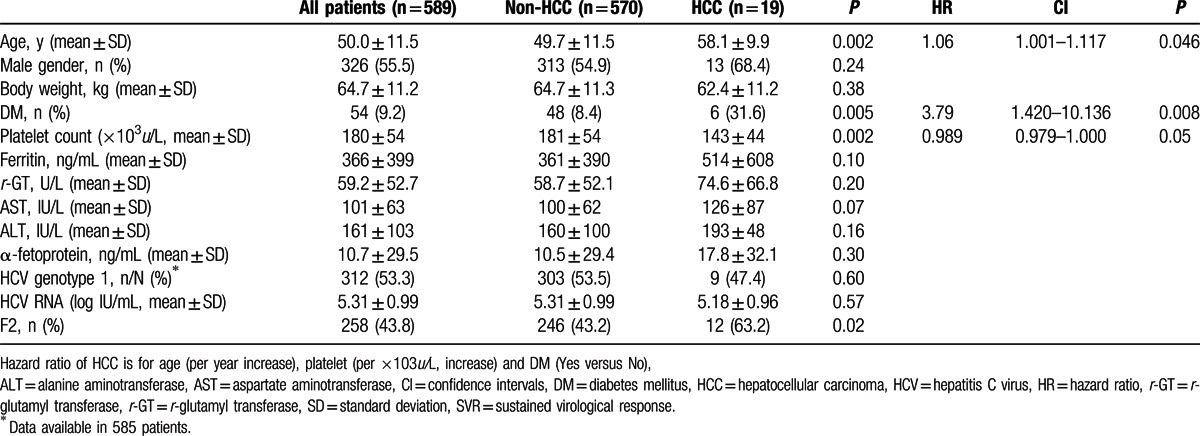
Factors associated with HCC development in SVR patients with mild liver disease (F0–2).

### 3.4 Influence of pretreatment and post-treatment glucose status in HCC development in SVR patients with mild liver disease

**Figure 2:**
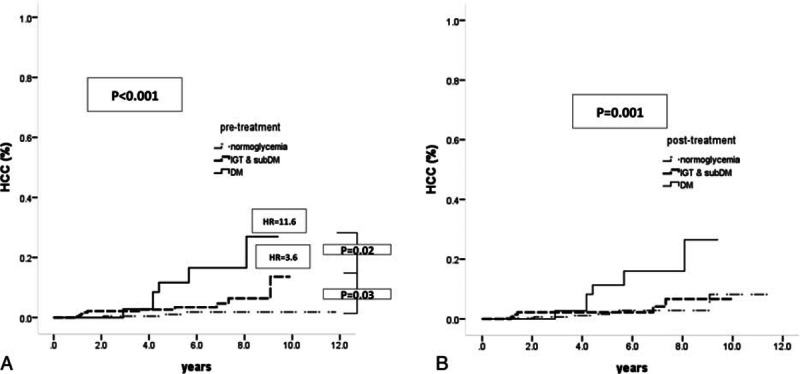
Risk and incidence of HCC development in SVR patients with mild liver disease are associated with glucose status before and after antiviral therapy: (A) before treatment; (B) after treatment. HCC = hepatocellular carcinoma, SVR = sustained virological response.

DM has a significant impact on HCC development in SVR patients with mild liver disease. We further explored the association of dynamic change singlucose status with HCC occurrence in this population. The proportions of patients with normoglycemia, pre-DM, sub-DM (pre-sDM), and DM before treatment were 45.3% (n = 267), 29.9% (n = 176), 15.6% (n = 92), and 9.2% (n = 54), respectively. The proportions of HCC in patients with normoglycemia, pre-sDM, and DM before treatment were 1.1%, 3.7%, and 11.1%, respectively (trend *P* < 0.001). Sixteen of 19 (84.2 %) HCC patients possessed glucose abnormalities (including 6 patients with DM and 10 patients with pre-sDM) before antiviral therapy. Compared to patients with normoglycemia, the incidence of HCC increased gradually from pre-sDM (HR: 3.6, *P* = 0.05) to DM (HR: 11.6, *P* = 0.001) (adjusted trend *P* = 0.004) (Table 3 and Fig. 2A).

**Table 3:**
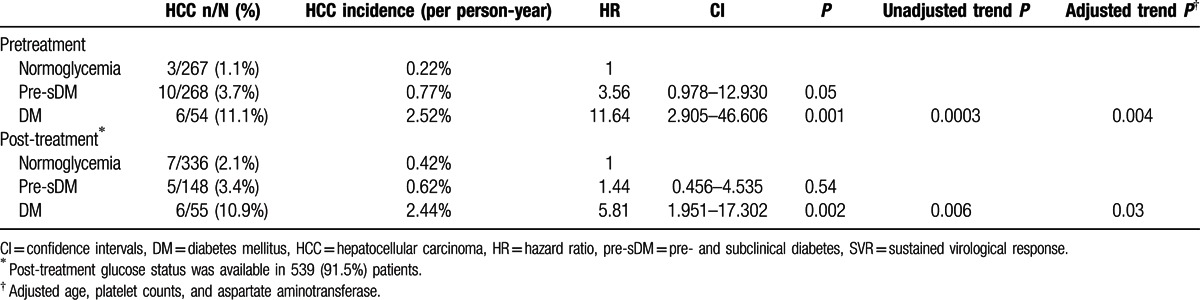
Incidence and risk of HCC development in SVR patients and mild liver disease with different pretreatment and post-treatment glucose status.

In total, 539 of the 589 (91.5%) patients had post-treatment glucose status information available. Of these patients, the proportions with normoglycemia, pre-DM, pre-sDM, and DM were 62.3% (n = 336), 20.6% (n = 111), 6.9% (n = 37), and 10.2% (n = 55), respectively. The rates of HCC in patients with normoglycemia, pre-sDM, and DM after treatment were 2.1%, 3.4%, and 10.9%, respectively (trend *P* = 0.003). Compared to normoglycemic patients, patients with DM were at significantly higher risk for developing HCC (HR/CI: 5.81/1.951–17.302, *P* = 0.002). However, based on post-treatment glucose status, the risk of HCC did not differ between normoglycemia and pre-sDM patients (Table 3 and Fig. 2B). Although patients were divided into normoglycemia, pre-sDM, and DM, the differences in glucose status did not impact HCC development in the other 3 subpopulations (SVR & F34, non-SVR & F0–2, and non-SVR & F34) (Supplementary Figure 1).

### 3.5 Influence of glucose augmentation in HCC

Of the 236 patients who were normoglycemic before treatment, the percentages of normoglycemic, pre-sDM, and DM patients after treatment were 74.6% (n = 176), 25.0% (n = 59), and 0.4% (n = 1), respectively. Of the 249 patients with pre-sDM before treatment, the percentages of normoglycemic, pre-sDM, and DM patients after treatment were 64.3% (n = 160), 35.7% (n = 89), and 0%, respectively. In total, the percentages of non-DM patients with improved, stable, and worse glucose status were 33.0% (n = 160), 54.6% (n = 265), and 12.4% (n = 60), respectively. The rates of HCC in non-DM patients with improved, stable, and worse glucose status were 2.5%, 3.0%, and 0%, respectively (*P* = 0.49), and the incidence of HCC did not differ among the 3 groups (*P* = 0.36, Supplementary Figure 2).

## 4 Discussion

The association of DM with HCC has been widely discussed. To our knowledge, our study is the first to explore the impact of dynamic changes in glucose status on HCC occurrence in CHC patients receiving antiviral therapy. Here, we demonstrated that DM is a major risk factor for HCC occurrence among patients with mild liver fibrosis despite the benefit of viral eradication. Notably, prediabetic status may also carry some risk for developing HCC. Most of the SVR patients with mild liver disease who developed HCC had a glucose abnormality prior to HCV eradication, even if the glucose status was ameliorated by curative antiviral therapy.

CHC patients with glucose abnormalities are generally older, have more advanced liver disease,^[7]^ and are prone to experience treatment failure. Because these factors are critical determinants for HCC development, they may confound the association of DM with HCC in patients receiving antiviral therapy. Therefore, the magnitude of the effect of DM on the chance of developing HCC may have been masked by other potential risk factors after statistical adjustment.^[16,28]^ However, DM does increase the risk of HCC development inpatients when restricted to the low-risk population. Although the incidence of HCC is low in SVR patients with mild liver disease, HCC does occur in a minority of patients upon long-term follow-up. We identified DM as the most critical risk factor for developing HCC in these patients, with HCC risk being ∼4-fold greater in diabetes patients than in nondiabetic patients. Similarly, Hung et al cautioned that HCC occurrence may be increased in noncirrhotic patients who have achieved SVR.^[18]^ Almost all DM patients experience the prediabetes condition (i.e., IFG and/or IGT) before a definite diagnosis is confirmed. In addition to developing DM, the prediabetes condition has been suggested to carry a risk of cardiovascular disease.^[1,18]^ Therefore, we aimed to determine if prediabetes similarly impacted HCC. We found a trend between HCC development and the transition from normoglycemia to DM. Insulin resistance, regardless of DM status, has been linked with HCV-related HCC,^[29]^ implying the potential oncogenic role of hyperglycemia in hepatocarcinogenesis. Although the pathophysiological mechanism remains unclear, insulin resistance might influence hepatocarcinogenesis via several molecular pathways, such as phosphatase and tensin homolog (PTEN)/P13K/Akt and MAPK kinase (MAPKK).^[30]^ Glucose abnormality might also be related to dysregulation of the insulin-like growth factor (IGF) system and the type-IIGF receptor (IGF-IR) signaling pathway, which is important for HCC development.^[31]^ The knowledge of these pathways has important consequences in the goal of HCC treatment.^[32–35]^

DM is one of the most significant extra hepatic manifestations of HCV infection. Previously, it was shown that the prevalence of glucose abnormalities was 3 times greater in anti-HCV positive patients thanin anti-HCV-negative patients.^[36]^ Robust epidemiological evidence has also shown that HCV viremia, but not anti-HCV seropositivity alone, increased the association with type 2 DM.^[6]^ Glucose status might be uncovered in CHC patients. In individuals without a known DM history, CHC patients were at higher risk for developing DM and IGT than controls (odds ratio 3.3).^[26]^ Consistent with our previous reports, without OGTT, 15.6% of the patients with subclinical DM would not have been identified. This finding reinforces the necessity of OGTT in CHC patients,^[37]^ not only for glucose management but also for long-term outcome surveillance. Glucose status and pancreatic beta-cell function may be augmented in patients receiving interferon-based antiviral therapy.^[14]^ Among nondiabetic SVR patients with mild liver disease, one-third had improved glucose status, and only one-tenth had deteriorated sugar after antiviral therapy. Notably, improvement in glucose status did not benefit HCC development, suggesting that insulin resistance elicited certain oncogenic processes that were beyond the impact of virus and fibrogenesis. Given the high prevalence of glucose abnormality corresponding to hepatic and extra hepatic long-term outcome in CHC patients, new parameters or cut off levels for defining glucose abnormality for normoglycemia, prediabetes and DM might be warranted.^[37]^ In conclusion, although the likelihood of developing HCC in CHC patients with mild fibrosis is low, it may still occur even after SVR is achieved. A major risk factor for this population is glucose abnormality. Due to their increased risk for HCC, patients with pre-sDM should undergo increased surveillance in the post-treatment period.

## Supplementary Material

Supplemental Digital Content
