# Supplementary material for: Pretreatment glucose status determines HCC development in HCV patients with mild liver disease after curative antiviral therapy
Source: Medicine (Baltimore). 2016 Jul 8;95(27):e4157. doi: 10.1097/MD.0000000000004157 (PMC5058864; doi:10.1097/MD.0000000000004157)

**Supplementary figure 1.**Differences in glucose status influence HCC development in three patient subpopulations (SVR & F34, non-SVR & F0-2, and non-SVR & F34)


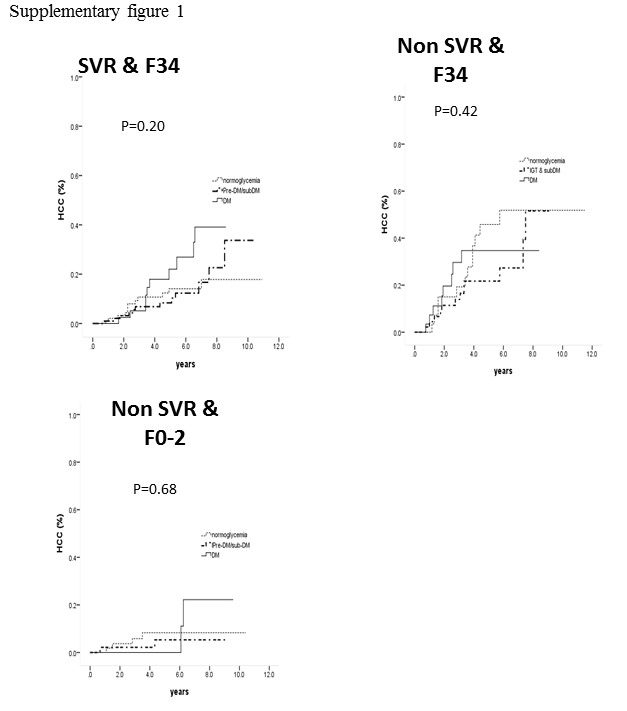


**Supplementary figure 2.** Risk of HCC development in SVR patients with mild liver disease based on different changes in glucose status


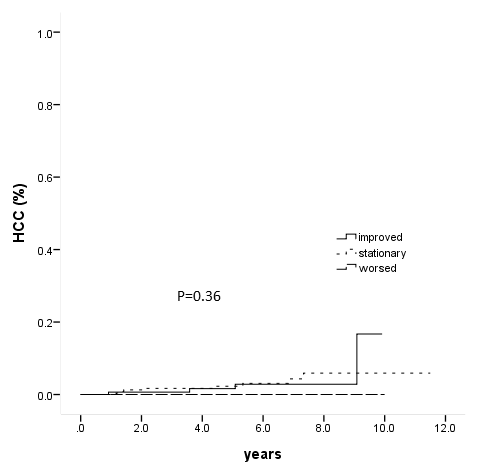

Supplement: Supplemental Digital Content [file medi-95-e4157-s001.doc]
